# Dietary Allicin Improved the Survival and Growth of Large Yellow Croaker (*Larimichthys crocea*) Larvae via Promoting Intestinal Development, Alleviating Inflammation and Enhancing Appetite

**DOI:** 10.3389/fphys.2020.587674

**Published:** 2020-10-09

**Authors:** Wenxing Huang, Chuanwei Yao, Yongtao Liu, Ning Xu, Zhaoyang Yin, Wenxuan Xu, Youqing Miao, Kangsen Mai, Qinghui Ai

**Affiliations:** ^1^Key Laboratory of Aquaculture Nutrition and Feed (Ministry of Agriculture and Rural Affair), Key Laboratory of Mariculture (Ministry of Education), Ocean University of China, Qingdao, China; ^2^Laboratory for Marine Fisheries Science and Food Production Processes, Qingdao National Laboratory for Marine Science and Technology, Qingdao, China

**Keywords:** *Larimichthys crocea*, larvae, allicin, growth, immunity, inflammation, appetite-related gene

## Abstract

A 30-day feeding experiment was conducted to investigate effects of dietary allicin on survival, growth, antioxidant capacity, innate immunity and expression of inflammatory and appetite related genes in large yellow croaker larvae. Four iso-nitrogenous (53% crude protein) and iso-lipidic (19% crude lipid) diets were formulated via supplementing graded levels of allicin (0.0 (the control), 0.005, 0.01, and 0.02% dry diet, respectively). Results showed that, among dietary treatments, larvae fed the diet with 0.005% allicin had the highest survival rate (SR) (*P* < 0.05), while larvae fed the diet with 0.01% allicin had the highest specific growth rate (SGR) (*P* < 0.05). Activities of α-amylase in both pancreatic (PS) and intestine segments (IS) of larvae fed the diet with 0.01% allicin were significantly lower than that in the control (*P* < 0.05). On the other hand, the supplementation of 0.01% allicin in diets significantly increased activities of alkaline phosphatase (AKP) and leucine aminopeptidase (LAP) in the intestinal brush border membrane (BBM) of larvae than the control (*P* < 0.05), indicating the promoting roles of allicin on fish larval intestinal development. Moreover, compared to the control, both the nitric oxide (NO) content and the activity of nitric oxide synthase (NOS) were significantly up-regulated in larvae fed the diet with 0.005% allicin, and catalase (CAT) were significantly upregulated in larvae fed the diet with 0.02% allicin (*P* < 0.05). Transcriptional levels of pro-inflammatory genes including cyclooxygenase-2 (*cox-2*), interleukin-1β (*il-1*β) and interleukin-6 (*il-6*) significantly decreased with increasing allicin, compared to the control. The expression of appetite genes including *npy*, *ghrelin* and *leptin* significantly increased with the prolonged fasting period, and dietary allicin supplementation significantly increased the transcriptional level of neuropeptide Y (*npy*) at 0.01%, while increased the transcriptional level of *leptin* in larvae at 0.02% dosages (*P* < 0.05). These results showed that the supplementation of 0.005% – 0.01% allicin in diets could improve the survival and growth of large yellow croaker larvae probably by promoting intestinal development, alleviating inflammation and enhancing appetite.

## Introduction

In recent years, many natural products from plants, animals and microorganisms have been shown to possess potential medicinal value for their extensive physiological function (Yuan et al., 2016). In particular, phytogenic compounds contain a variety of bioactive ingredients, of which the higher proportion of component determines their main biological characteristic (Santos et al., 2011; Chakraborty et al., 2014). Some active phytogenic compounds, such as thymol, carvacrol, limonene, cinnamaldehyde and eugenol from the plant thyme, oregano, citrus, cinnamon and clove respectively, have been noted to exert positive effects on nutrition and health of animals (Wallace et al., 2010; Chakraborty et al., 2014; Upadhaya and Kim, 2017; Sutili et al., 2018). Garlic (*Allium sativum*) has been reported with multiple positive effects on animals, such as decreasing cholesterol and fatty acid levels in the blood (Eilat et al., 1995; Abramovitz et al., 1999), easing blood pressure (Silagy and Neil, 1994; Turner et al., 2004; Benavides et al., 2007; Ried et al., 2008) and preventing cardiovascular disease (Chan et al., 2013). Allicin is the predominant bioactive compound in fresh garlic, which represents about 70% of total thiosulfinates existing in crushed garlic (Calvey et al., 1994) and gives the garlic its characteristic smell (Kumar and Berwal, 1998). Allicin from the extract of raw garlic showed antibacterial activity (Leontiev et al., 2018), and meanwhile, the effects of allicin as an antifungal, antiprotozoa and antiviral agent also have been investigated (Curtis et al., 2004; Fujisawa et al., 2009). Besides the anti-microorganims roles, allicin also functions as the immune stimulant in rat (Patya et al., 2004; Toma et al., 2019), mice (Bruck et al., 2005; Feng et al., 2012; Chen et al., 2019) and pig (Lan et al., 2017). Recently, allicin is also reported to improve the non-specific immunity and disease resistance of fish (Jeney and Jeney, 2002; Petrunov et al., 2007; Aly and Mohamed, 2010; Nya and Austin, 2011; Talpura and Ikhwanuddin, 2012).

Compared to juvenile and adult fish, fish larvae are relatively fragile and susceptible to environmental stress due to the incomplete tissue development and immune system (Cai et al., 2016), and thus high mortality rate always occurs during the larval culture period (Rojo-Cebreros et al., 2018). Large yellow croaker (*Larimichthys crocea*) is a carnivorous marine fish widely cultivated in southeastern China, with the largest production among all marine fish species in China, due to its delicious taste (Ai et al., 2008; Xie et al., 2011; Zhao et al., 2013; Liu et al., 2020). However, high mortality still exists in large yellow croaker larvae period. Considering the possibility of chemical residents when using chemicals to treat fish larvae mortality, it is promising to improve fish immunity via nutritional modulation. Fish larvae always undergo significant physiological changes during the early developmental stages, and their feeding biology and ecology also experienced profound changes at the same time (Ronnestad et al., 1999; Opazo et al., 2017). To date, a few preliminary studies have evaluated the nutritional requirement, feeding habits and histological development of the digestive system in large yellow croaker larvae (Mai et al., 2005; Ai et al., 2008; Xie et al., 2011; Yu et al., 2012; Zhao et al., 2013; Cai et al., 2015, 2016; Feng et al., 2017; Liu et al., 2020). It is of great importance to evaluate the influence of dietary supplementation of phytogenic compounds on the digestive system development, immunity and appetite of large yellow croaker larvae. Therefore, the present study determined the effects of allicin supplementation on the growth, survival, antioxidant capacity, innate immunity, inflammation and appetite of large yellow croaker larvae. To our knowledge, this is the first study to evaluate the regulatory mechanism of dietary allicin supplementation on survival and growth of marine fish larvae.

## Materials and Methods

### Feed Ingredients and Diets Formulation

The experimental diets were formulated based on the nutrient requirements of large yellow croaker larvae according to Ai et al. (2008) with some modification. White fishmeal (crude protein, 71.73%), krill meal (64.86%), squid meal (81.81%) and soy protein concentrate (66.70%) were used as protein sources. Fish oil and soybean lecithin were used as lipid sources. Microcrystalline cellulose was used to maintain the balance of each treatment group. Ingredients were purchased from Guangdong VTR Bio-Tech Co., Ltd. (Zhuhai, China) and Qingdao Bio-ways Ingredients Biotechnology Co., Ltd. (Qingdao, China). Four iso-nitrogenous (53% crude protein) and iso-lipidic (19% crude lipid) experimental diets were formulated and supplementation with 0.0 (the control), 0.005, 0.01, and 0.02% of allicin, respectively (Table 1). Pure allicin (over 98%) was purchased from Shanghai Yuanye Bio-Technology Co., Ltd., in Shanghai, China.

**TABLE 1:** Formulation and proximate analysis of the experimental diets (% dry matter).

Ingredients	Diets (allicin supplementation level%)			
	
	Diet1 (0%)	Diet2 (0.005%)	Diet3 (0.01%)	Diet4 (0.02%)
Whitefish meal ^a^	36	36	36	36
Krill meal ^b^	10	10	10	10
Squid meal^c^	4	4	4	4
Soy protein concentrate ^b^	5	5	5	5
Wheat gluten ^b^	12	12	12	12
Yeast	4	4	4	4
microcrystalline celluloseto	2	1.995	1.99	1.98
α-Starch	8	8	8	8
Alginae sodium	2	2	2	2
Vitamin premix ^d^	1.5	1.5	1.5	1.5
Mineral premix ^e^	1	1	1	1
Ascorbyl polyphosphate	0.2	0.2	0.2	0.2
Mold inhibitor	0.05	0.05	0.05	0.05
Antioxidant	0.05	0.05	0.05	0.05
Choline chloride	0.2	0.2	0.2	0.2
Fish oil	9	9	9	9
Soybean lecithin	5	5	5	5
Allicin ^f^	0	0.005	0.01	0.02
Proximate composition				
Crude protein	53.72	53.32	53.71	53.46
Crude lipid	18.36	19.05	18.80	18.99
Ash	12.86	12.04	12.10	12.05

*^*a*^Commercially available from Guangdong VTR Bio-Tech Co., Ltd. (Zhuhai, China); elementary composition (dry matter): White fish meal, crude protein, 71.73%, crude lipid,4.76%. ^*b*^Commercially available from Qingdao Bio-ways Ingredients Biotechnology Co., Ltd. (Qingdao, China); elementary composition (dry matter): Krill meal, crude protein, 64.86%, crude lipid, 8.0%. Soy protein concentrate, crude protein, 66.70%. Wheat gluten, crude protein, 85.06%. ^*c*^Commercially available from Haixingyuan Feed Co., Ltd., Co., Ltd. (Hebei, China); elementary composition (dry matter): Squid meal, crude protein, 81.81%, crude lipid, 5.16%. ^*d*^Composition of vitamin premix (IU or g ^*kg*–1^): vitamin A palmitate, 3000000 IU; vitamin D_3_, 1200000 IU; DL-α-vitamin E, 40.0 g kg^–1^; menadione, 8.0 g kg^–1^; thiamine-HCl, 5.0 g kg^–1^; riboflavin, 5.0 g kg^–1^; D-calcium pantothenate, 16.0 mg kg^–1^; pyridoxine-HCl, 4.0 mg kg^–1^; inositol, 200.0 mg kg^–1^; biotin, 8.0 mg kg^–1^; folic acid, 1.5 mg kg^–1^; 4-aminobenzoic acid, 5.0 mg kg^–1^; niacin, 20.0 mg kg^–1;^vitamin B_12_, 0.01 mg kg^–1^; L-ascorgyl-2-monophosphate-Na (3%),2000.0 mg kg^–1^. ^*e*^Composition of mineral premix (g kg^–1^ premix): Ca(H_2_PO_4_)⋅H_2_O,675.0; C_*O*_SO_4_⋅H_2_O, 0.15; CuSO_4_⋅H_2_O, 5.0; FeSO_4_⋅7H_2_O, 50.0; KCl, 0.1; MnSO_4_⋅2H_2_O, 101.7; MnSO_4_⋅2H_2_O, 18.0; NaCl, 80.0; NaSeO_3_⋅H_2_O, 0.05; ZnSO_4_⋅7H_2_O, 20.0. ^*f*^The purity of allicin was over 98%, purchased from Shanghai yuanye Bio-Technology Co., Ltd., in Shanghai, China.*

Micro-diet (MD) was manufactured by micro-bonding technology. All ingredients were firstly ground to a fine powder through a 150 μm nylon mesh. Diets were prepared after thoroughly mixing dry ingredients with fish oil, phospholipid oil and water. The pellets were dried at 50°C in a constant temperature oven for 8 h. The dry pellets were ground into 250–380 μm and 380–500 μm particle sizes, and subsequently stored at −20°C until used. The larvae were fed 250–380 μm diets between 15 and 25 days after hatch (DAH), and then 380–500 μm diets.

### Fish and Experimental Procedures

Large yellow croaker larvae used in the present study were obtained from Xiangshan Harbor Aquatic Seeds Company, Ningbo, China and reared at Marine and Fishery Science and Technology Innovation Base, Ningbo, China.). All larvae in the hatchery were fed with rotifers, Brachionus plicatilis (0.5 – 1.5 × 104 individuals/l) from 3 to 8 days after hatch (DAH), Artemia nauplii (1.0 – 1.5 × 103 individuals/l) from 6 to 11 DAH, and live copepods from 10 to 14 DAH. Fish larvae were fed with a mixture of live copepods and artificial micro-diet from 15 to 16 DAH to adapt to the experimental diet. The experiment was carried out in 12 white plastic tanks (water volume 220L), where four experimental diets were randomly allocated to triplicate groups of larvae and each tank was stocked with a density of 3500 larvae. During the experiment, the water temperature was maintained at 21.0–23.0°C, the salinity was ranged from 25.0 to 28.0 g/L, and the dissolved oxygen was above 6.0 mg/L. About 100–200% of the water volume was renewed daily. The feeding experiment lasted for 30 days, during which fish larvae were fed with 250–380 μm diets from15 to 25 DAH, and then 380–500 μm diets after 25 DAH.

### Sampling and Dissection

At the end of the experiment, forty individuals from each tank were sampled at 1, 3, 6, 12, and 24 h to analyze the expressions of appetite genes. One hundred individuals from each tank were sampled after fasting for 24 h and immediately frozen in liquid nitrogen and then stored at −80°C for enzymatic and inflammatory genes expression assay. The remaining fish from each tank were collected and stored at −20°C for body composition analysis.

The intestine segment (IS), pancreatic segments (PS), head, and visceral mass of fish larvae were separated under a dissecting microscope and placed on a glass plate which was kept at 0°C for digestive enzyme activity analysis as early described Cahu and Infante (1994). Fifty larvae at 15 DAH were randomly sampled to measure the initial body weight. After the feeding experiment, the final body weight (FBW) of fifty individuals per tank was measured with a microbalance. Initial body length (IBL) and final body length (FBL) were measured with a vernier caliper, and the survival rate was determined by counting the individuals remaining in each tank.

### Analytical Methods for Enzymes

The samples of diets and fish larvae were dried to a constant weight at 105°C to determine their dry matter. Crude lipid and crude protein of ingredients, diets and fish carcass were measured following the standard methods of the Association of Official Analytical Chemists (AOAC International, 1995). Intestinal segments (0.2–0.3 g) of fish larvae were homogenized to purify brush border membranes (BBM) according to a method for intestinal scrapping (Crane et al., 1979) and adapted to intestinal segments (Cahu and Infante, 1994), and 1 ml homogenate was removed for intestinal enzyme assays. This homogenate was then centrifuged at 3300 g for 3 min and the supernatant was used for enzyme assays. Activities of catalase (CAT), total antioxidant capacity (T-AOC) and the content of malondialdehyde (MDA) in visceral mass were determined by commercial reagents and kits (Nanjing Jiancheng Bio-Engineering Institute, China). Activities of lysozyme (LZ), nitric oxide synthase (include TNOS, iNOS and eNOS), superoxide dismutase (include T-SOD, CuZn-SOD and Mn-SOD) and the content of nitric oxide (NO) in visceral mass were determined by commercial reagents and kits (Nanjing Jiancheng Bio-Engineering Institute, China). Activities of α-amylase, trypsin and lipase in intestinal segments (PS) and pancreatic segments (IS), leucine aminopeptidase (LAP), and alkaline phosphatase activity (AKP) in the brush border membrane (BBM) of fish larval intestine were determined by commercial reagents and kits (Nanjing Jiancheng Bio-Engineering Institute, China) according to the instructions (Liu et al., 2020).

### Total RNA Extraction, cDNA Synthesis and Real-Time Quantitative Polymerase Chain Reaction (RT-qPCR)

Total RNA was extracted from fish larvae head and visceral mass using TRIzol reagent (Takara, Japan), and then electrophoresed on a 1.2% denaturing agarose gel to detect the quality. The quantity of extracted RNA was detected by a NanoDrop spectrophotometer (Thermo Scientific, United States). The extracted RNA was reversed to cDNA with the PrimeScript^TM^ RT reagent Kit (Takara, Japan) following the manufacturer’s instructions. The β*-actin* was considered as the housekeeping gene in the present study (Li et al., 2019). The RT-qPCR primers of the candidate genes were designed based on the nucleotide sequences of large yellow croaker (Table 2). The amplification was performed in a total volume of 25 μL containing 1 μL of each primer, 1 μL of cDNA, 12.5 μL of SYBR Premix Ex Taq II (Takara, Japan), and 9.5 μL of RNase-free water. The RT-qPCR program was as follows: 95°C for 2 min, followed by 40 cycles of 95°C for 10 s, 57°C for 10 s, and 72°C for 20 s. Melting curve analysis was carried to confirm that a single PCR product was present in these reactions at the end of the PCR reaction. Standard curves were prepared with 4-fold serial dilutions (in triplicate) of cDNA which was used to calculate the amplification efficiency by the following equation: E = 10^(–1/slope)^ −1. The amplification efficiencies of the target and reference genes primers ranged between 0.95 and 1.05. The gene expression levels were calculated with the 2^–Δ^ ^Δ^ ^*CT*^ method as described by Livak and Schmittgen (2001).

**TABLE 2:** Sequences of the PCR primers used in this study.

Target gene	Forward primers (5′-3′)	Reverse primers (5′-3′)	Accession number
*npy*	AAAGAGGTCCAGTCCTGAGATT	GTGGCGGCTCATAGTGGTAA	XM019275410
*ghrelin*	TGACCTTGTGGTGCAAGTCGAC	CCGATTTCAAAGGGGGCACT	KC899122
*leptin*	GATGTTCTGGATGGACCTGC	AGACACCACTGATGCGGACT	XM010729765
*tnfα*	ACACCTCTCAGCCACAGGAT	CCGTGTCCCACTCCATAGTT	NM001303385
*ifn*γ	TCAGACCTCCGCACCATCA	GCAACCATTGTAACGCCACTTA	XM019258900
*cox-2*	CTGGAAAGGCAACACAAGC	CGGTGAGAGTCAGGGACAT	XM010734489
*il-1*β	CATAGGGATGGGGACAACGA	AGGGGACGGACACAAGGGTA	XM010736551
*il-6*	CGACACACCCACTATTTACAAC	TCCCATTTTCTGAACTGCCTCT	XM010734753
β*-actin*	GACCTGACAGACTACCTCATG	AGTTGAAGGTGGTCTCGTGGA	GU584189

**Npy*, neuropeptide Y; *tnfα*, tumor necrosis factor *α*; *ifn*γ, interferon γ; *cox-2*, cyclooxygenase-2; *il-1*β, interleukin-1β; *il-6*, interleukin-6.*

### Calculations and Statistical Analysis

Survival rate (SR,%) = 100N_t_/N_0_

Specific growth rate (SGR,% day-1) = 100(Ln W_t_ - Ln W_0_)/d

Where N_t_ and N_0_ were final and initial fish numbers; W_t_ was the final wet body weight (g), W_0_ was the initial wet body weight and d was the experimental duration, respectively.

All data, excepting appetite genes expression data, was subjected to a one-way ANOVA using the software SPSS 19.0 (IBM, America). The fish appetite genes expression data were analyzed by two-way ANOVA (diet × time) to determine the effects of diet, fasting time, and interaction. Differences in expression of different time points under the same diet and different diets at the same time were subjected to a one-way ANOVA. Statistics with *P* < 0.05 were regarded to be significant, and the results were shown as means ± S.E.M. (standard error of the mean).

## Results

### Effects of Dietary Allicin on Survival, Growth, and Body Composition of Fish Larvae

Larvae survival rate (SR) showed a trend to firstly increase and then decrease with increasing dietary allicin dosage. The survival rates of larvae fed diets with 0.005% allicin (21.36%) and 0.01% allicin (20.26%) were significantly higher than that of the control larvae (15.01%) (*P* < 0.05). Specific growth rate (SGR) of larvae also showed the similar trend and the specific growth rate of larvae fed the diet with 0.01% allicin (8.93% day^–1^) was significantly higher than the control (8.38% day^–1^) (*P* < 0.05). Similar results were also found in final body length (FBL) and final body weight (FBW) of fish larvae (Table 3). No significant difference in the protein, lipid, and moisture of larvae whole body were found among different dietary treatments (*P* > 0.05) (Table 4).

**TABLE 3:** Effects of dietary allicin on survival and growth performance of large yellow croaker post larvae (Means ± S.E.M)^a^.

Index	Experimental diets (allicin level)			
	
	Diet1 (0%)	Diet2 (0.005%)	Diet3 (0.01%)	Diet4 (0.02%)
Final body length (FBL, mm)	13.92 ± 0.34^b^	15.31 ± 0.45^ab^	15.82 ± 0.57^a^	15.18 ± 0.22^ab^
Final body weight (FBW, mg)	58.23 ± 1.36^b^	62.36 ± 1.43^ab^	68.64 ± 1.53^a^	57.88 ± 1.29^b^
Specific growth rate (SGR,% day^–1^)	8.38 ± 0.08^b^	8.60 ± 0.08^ab^	8.93 ± 0.07^a^	8.36 ± 0.08^b^
Survival rate (SR,%)	15.01 ± 1.05^b^	21.36 ± 1.00^a^	20.26 ± 1.06^a^	18.46 ± 0.42^ab^

*^*a*^Data are presented as means ± S.E.M. Means in each row sharing the same superscript letter or absence of superscripts are not significantly different determined by Tukey’s test (*P* > 0.05). S.E.M.: standard error of means.*

**TABLE 4:** Body composition of large yellow croaker post larvae (Means ± S.E.M)^a^.

Whole-body (%)	Experimental diets			
	
	Diet1 (0%)	Diet2 (0.005%)	Diet3 (0.01%)	Diet4 (0.02%)
Protein	6.01 ± 0.08	5.81 ± 0.13	5.54 ± 0.06	5.41 ± 0.19
Lipid	2.45 ± 0.14	2.37 ± 0.18	2.16 ± 0.17	2.36 ± 0.08
Moisture	89.19 ± 0.71	89.48 ± 0.39	89.78 ± 0.53	89.99 ± 0.23

*^*a*^Data are presented as means ± S.E.M. Means in each row sharing the same superscript letter or absence of superscripts are not significantly different determined by Tukey’s test (*P* > 0.05). S.E.M.: standard error of means.*

### Effects of Dietary Allicin on Specific Activities of Digestive Enzyme of Fish Larvae

**FIGURE 1:**
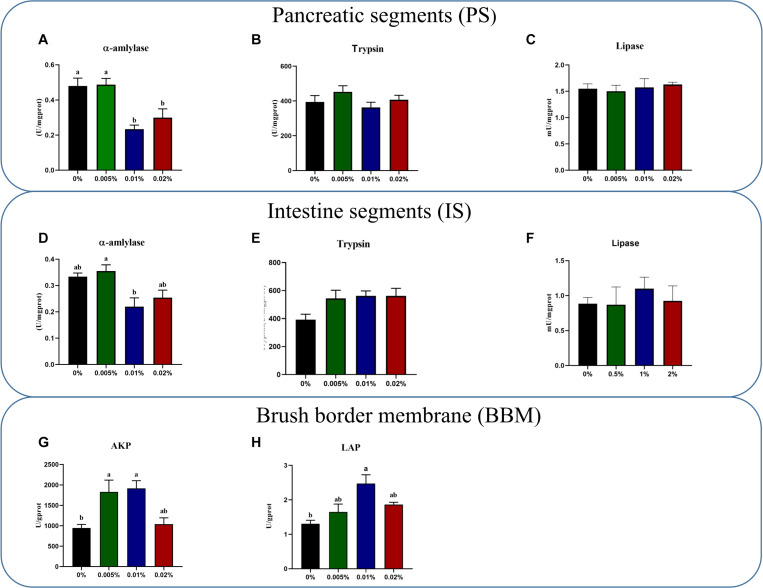
The specific activities of α-amylase **(A)**, trypsin **(B)** and lipase **(C)** in pancreatic segments. The specific activities of α-amylase **(D)**, trypsin **(E)** and lipase **(F)** in intestinal segments **(B)**. The specific activity of AKP **(G)** and LAP **(H)** on the brush border membrane in the large yellow croaker larvae. AKP, alkaline phosphatase; LAP, leucine aminopeptidase. Data are presented as means ± S.E.M. Columns sharing the same superscript letter or absence of superscripts are not significantly different determined by Tukey’s test (*P* > 0.05). S.E.M.: standard error of means.

The activity of α-amylase in both larval PS and IS decreased significantly with increasing dietary allicin. The activity of α-amylase in larvae PS fed diets with 0.01% and 0.02% allicin was significantly lower than larvae fed the diet with 0.005% allicin and the control group (*P* < 0.05) (Figure 1A). The activity of α-amylase in larval IS fed the diet with 0.01% allicin was significantly lower than larvae fed the diet with 0.005% allicin (*P* < 0.05) (Figure 1D). However, no significant difference was observed in the activity of trypsin and lipase in larval PS and IS, respectively (*P* > 0.05) (Figures 1B,C,E,F).

Activities of AKP and LAP in larval brush border membranes (BBM) showed a trend to firstly increased and then decreased with increasing dietary allicin. Specifically, the activity AKP in larvae fed diets with 0.005% and 0.01% allicin was significantly higher (*P* < 0.05) (Figure 1G), while the activity of LAP in larvae fed the diet with 0.01% allicin was significantly higher (*P* < 0.05) (Figure 1H).

### Effects of Dietary Allicin on Antioxidant and Innate Immunity Capacity of Fish Larvae

**FIGURE 2:**
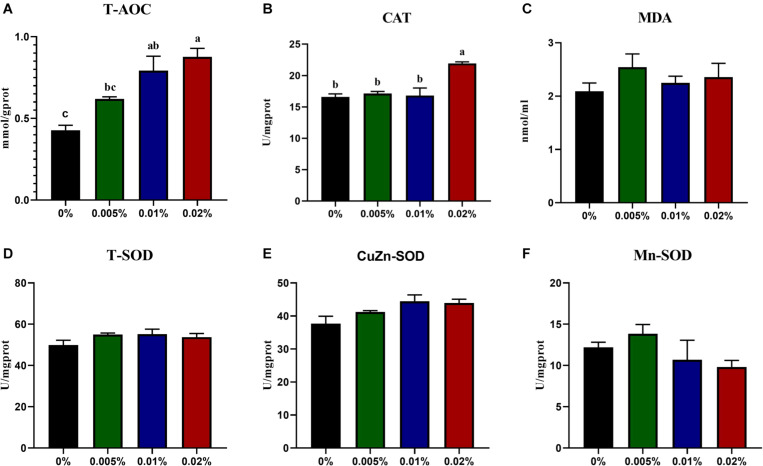
The specific activity of T-AOC **(A)**, CAT **(B)**, Concentration of MDA **(C)**, The specific activity of T-SOD **(D)**, CuZn-SOD **(E)** and Mn-SOD **(F)** in the visceral mass of large yellow croaker larvae. T-AOC, Total antioxidant capacity; CAT, catalase; MDA, malondialdehyde; T-SOD, total superoxide dismutase. Data are presented as means ± S.E.M. Columns sharing the same superscript letter or absence of superscripts are not significantly different determined by Tukey’s test (*P* > 0.05). S.E.M.: standard error of means.

The activity of T-AOC showed an increasing trend with increasing dietary allicin, and larvae fed diets with 0.005, 0.01, and 0.02% allicin was significantly higher than the control group (*P* < 0.05) (Figure 2A). Also, the activity of CAT in larvae fed the diet with 0.02% allicin was significantly higher than the control group (*P* < 0.05) (Figure 2B). However, the concentration of MDA and activities of SOD (include T-SOD, CuZn-SOD and Mn-SOD) showed no significant difference among dietary treatments (*P* > 0.05) (Figures 2C–F).

**FIGURE 3:**
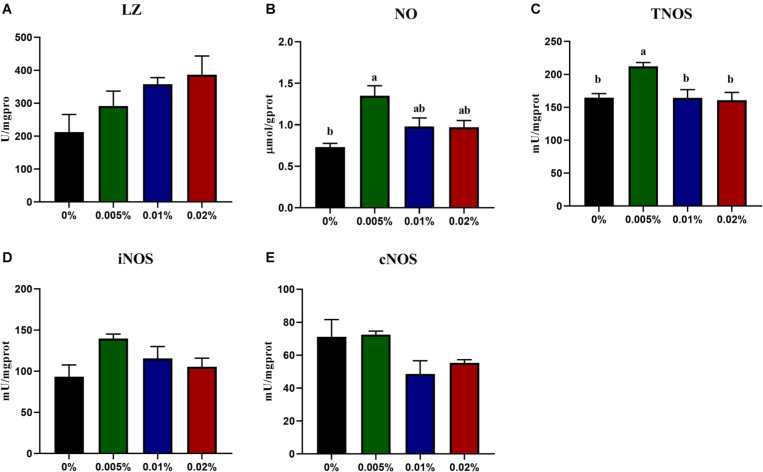
The specific activity of LZ **(A)**, the content of NO **(B)**, specific activity of TNOS **(C)**, iNOS **(D)** and cNOS **(E)** in the visceral mass of large yellow croaker larvae. LZ, lysozyme; NO, nitric oxide; TNOS, total nitric oxide synthase. Data are presented as means ± S.E.M. Columns sharing the same superscript letter or absence of superscripts are not significantly different determined by Tukey’s test (*P* > 0.05). S.E.M.: standard error of means.

**FIGURE 4:**
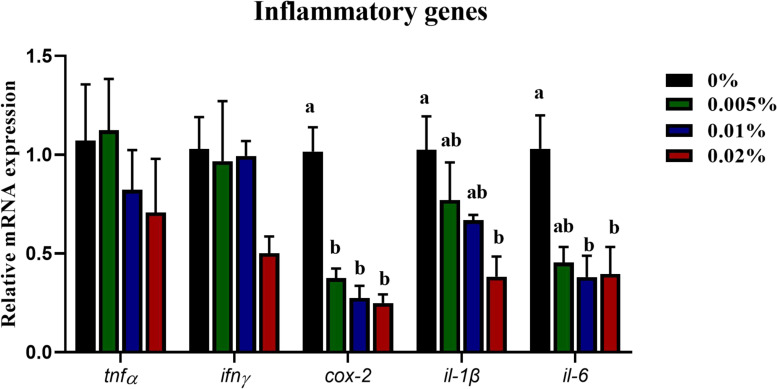
Expression of genes related to inflammation in the visceral mass of large yellow croaker larvae. *Tnfα*, tumor necrosis factor α; *ifn*γ, interferon γ; *cox-2*, cyclooxygenase-2; *il-1*β, interleukin-1β; *il-6*, interleukin-6. Data are presented as means ± S.E.M. Columns sharing the same superscript letter or absence of superscripts are not significantly different determined by Tukey’s test (*P* > 0.05). S.E.M.: standard error of means.

The innate immunity of fish larvae was assayed, including the content of NO, NOS enzyme activity and cytokines expression. The LZ activity showed an increasing trend with increasing dietary allicin, however, no statistical difference was observed among dietary treatments (*P* > 0.05) (Figure 3A). Dietary allicin supplementation increased the content of NO and the activity of TNOS in larvae, and both NO content and NOS activity in larvae fed the diet with 0.005% allicin was significantly higher than the control group (*P* < 0.05) (Figures 3B,C). However, the isotypes of NOS include iNOS and cNOS showed no significant difference among four dietary treatments (*P* > 0.05) (Figures 3D,E). Besides the enzyme activity, the expression of pro-inflammatory cytokines was also analyzed. The transcriptional levels of tumor necrosis factor α (*tnfα*) and interferon γ (*ifn*γ) in larvae showed no significant differences among dietary treatments (*P* > 0.05). The transcriptional level of cyclooxygenase-2 (*cox-2*), interleukin-1β (*il-1*β) and interleukin-6 (*il-6*) decreased significantly with the increasing dietary allicin. Transcriptional level of *cox-2* in larvae fed diets with 0.005, 0.01, and 0.02% allicin was significantly lower than the control group (*P* < 0.05). The transcriptional level of *il-1*β in larvae fed the diet with 0.02% allicin was significantly lower than the control group (*P* < 0.05). The transcriptional level of *il-6* in larvae fed diets with 0.01% and 0.02% allicin was significantly lower than the control group (*P* < 0.05) (Figure 4).

### Effects of Dietary Allicin on Appetite Genes Expression of Fish Larvae

**FIGURE 5:**
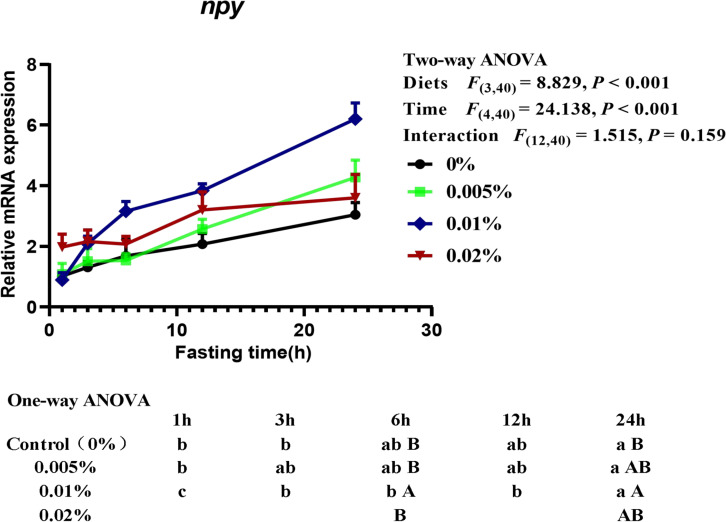
Relative mRNA expression of *npy* in the head of large yellow croaker larvae. Using Tukey’s multiple comparison test, different time points with different lowercase letters at the same diet and different diets with different capital letters at the same time point indicate significant differences (*P* < 0.05). Two-way ANOVA to compare the effects of short-term fasting on diet, time and interaction. The results were shown as means ± S.E.M. (standard error of the mean).

Two-way ANOVA (diet × time) showed that the transcriptional level of neuropeptide Y (*npy*) was significantly affected by both diet and fasting time (diets: *F*_(3,40)_ = 8.829, *P* < 0.001; time: *F*_(4,40)_ = 24.138, *P* < 0.001), although no interactive effects between fasting time and diets were found (interaction: *F*_(12,40)_ = 1.515, *P* = 0.159) (Figure 5). With the extension of fasting time, the transcriptional level of *npy* in larvae reached the highest level after fasting for 24-h. The transcriptional level of *npy* in larvae fed the diet with 0.005% allicin and the control group significantly increased after fasting for 24-h, while larvae fed the diet with 0.01% allicin significantly increased *npy* gene expression since fasting for 3-h (*P* < 0.05). However, the *npy* transcription level of larvae fed with 0.02% allicin diet did not reach a significant level within 24-h of fasting (*P* > 0.05). On the other hand, within specific time point, the transcriptional level of *npy* in larvae fed the diet with 0.01% allicin was significantly higher than that all other three groups after fasting for 6-hours, and also higher than the control group after fasting for 24-h (*P* > 0.05) (Figure 5).

**FIGURE 6:**
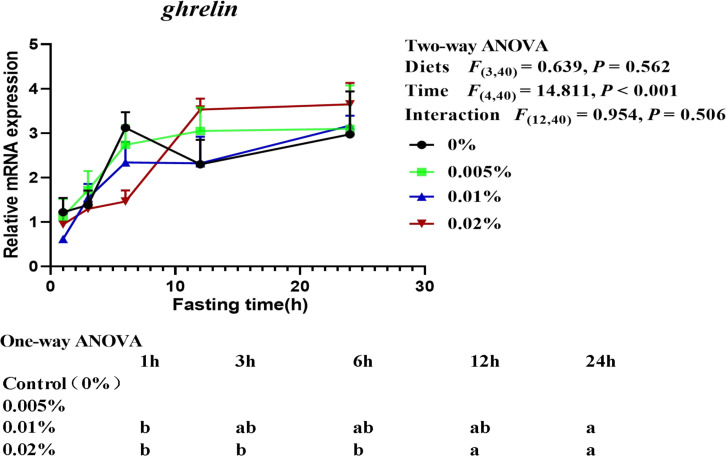
Relative mRNA expression of *ghrelin* in the visceral mass of large yellow croaker larvae. Using Tukey’s multiple comparison test, different time points with different lowercase letters at the same diet and different diets with different capital letters at the same time point indicate significant differences (*P* < 0.05). Two-way ANOVA to compare the effects of short-term fasting on diet, time and interaction. The results were shown as means ± S.E.M. (standard error of the mean).

The transcriptional level of *ghrelin* was significantly affected by fasting time (time: *F*_(4,40)_ = 14.811, *P* < 0.001), but not by diets and the interaction of diets and time (diets: *F*_(3,40)_ = 0.639, *P* = 0.562; interaction: *F*_(12,40)_ = 0.954, *P* = 0.506) (Figure 6). The transcriptional level of *ghrelin* in larvae fed the diet with 0.01% allicin was significantly increased since fasting for 12-h, while the transcriptional level of *ghrelin* in larvae fed the diet with 0.02% allicin significantly increased after fasting for 24-h (*P* < 0.05). However, the transcriptional level of *ghrelin* in larvae fed the diet with 0% and 0.005% allicin showed no significant difference within 24-h of fasting (*P* > 0.05) (Figure 6).

**FIGURE 7:**
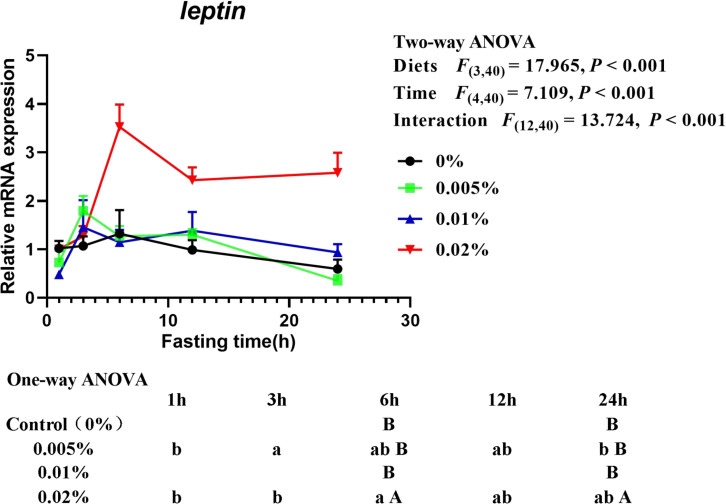
Relative mRNA expression of *leptin* in the visceral mass of large yellow croaker larvae. Using Tukey’s multiple comparison test, different time points with different lowercase letters at the same diet and different diets with different capital letters at the same time point indicate significant differences (*P* < 0.05). Two-way ANOVA to compare the effects of short-term fasting on diet, time and interaction. The results were shown as means ± S.E.M. (standard error of the mean).

The transcriptional level of *leptin* was significantly affected by diets, fasting time and interactive effects between diets and fasting time were also found (diets: *F*_(3,40)_ = 17.965, *P* < 0.001; time: *F*_(4,40)_ = 7.109, *P* < 0.001; interaction: *F*_(12,40)_ = 13.724, *P* < 0.001) (Figure 7). With the extension of fasting time, the transcriptional level of *leptin* increased at first and then decreased. Transcriptional level of *leptin* in larvae fed the diet with 0.005% allicin increased significantly after fasting for 3-h and then fell to a similar level after fasting for 24-h (*P* < 0.05), while larvae fed the diet with 0.02% allicin increased significantly after fasting for 6-h (*P* < 0.05) and then decreased. The transcriptional level of *leptin* in larvae fed the diet with 0% and 0.01% allicin showed no significant difference within 24-h of fasting (*P* > 0.05). On the other hand, within specific time point, the transcriptional level of *leptin* in larvae fed the diet with 0.02% allicin was significantly higher than the other three dietary treatments after fasting for 6-h and 24-h (*P* > 0.05) (Figure 7).

## Discussion

In the present study, dietary allicin at appropriate dosage (0.005–0.01% supplementation) could increase survival and growth of large yellow croaker larvae, which agrees well with some previous studies in other fish species including rainbow trout (Buyukdeveci et al., 2018), African catfish (Gabriel et al., 2019) and Asian sea bass (Talpura and Ikhwanuddin, 2012). Fish larvae underwent significant physiological changes, among which digestive system quickly developed and efficient digestion could promote growth and survival of fish larvae (Abiayad and Kestemont, 1994; Li et al., 2016; Liu et al., 2020). Fish digestive enzyme activity including α-amylase, lipase and trypsin in the intestine are significantly associated with fish digestive system development. Especially, the BBM enzymes (both AKP and LAP) in the intestine are important reporters to reflect the maturity of intestinal cells during larval development (Cahu et al., 1998). The reduced activity of α-amylase could be associated with the allometric growth of fish biomass (Infante and Cahu, 1994). Mai et al. (2005) also had elucidated the decline of amylase during the normal maturation process in large yellow croaker larvae. In the present study, the activity of α-amylase in larval PS and IS decreased significantly with the increase of dietary allicin level and the lowest activity was found in larvae fed the diet with 0.01% allicin, which was in consistent with findings in final body length, weight and SGR with the top value at 0.01% allicin. Furthermore, the activities of AKP and LAP significantly increased in larvae fed the diet with 0.01% allicin compared with the control, which indicated that dietary allicin might promote the development and maturation of fish larval intestine. Previous studies on rainbow trout and Japanese sea bass also showed that garlic extract could improve the digestion (Esmaeili et al., 2017; Xu et al., 2020). Therefore, the promoting roles of allicin on fish larvae intestinal development may also contribute to the higher survival and growth performance of large yellow croaker larvae.

The high energy consumption required for the rapid growth of marine fish larvae also makes them vulnerable to oxidative stress (Liu et al., 2015). Early studies have shown that allicin can protect osteoblasts from H_2_O_2_-induced oxidative stress (Ding et al., 2016), and allicin can scavenge oxygen free radicals and also affect the antioxidant capacity of animals (Chung, 2006). Purportedly, allicin protects cells against oxidative stress by inducing the production of antioxidant enzymes (El-Sheakh et al., 2016). Previous studies have shown that allicin supplementation improves oxidative stress by increasing activities of CAT and SOD and reducing MDA content in rats (Abdel-Daim et al., 2017), mice (Panyod et al., 2016) and Nile tilapia (Abdel-Daim et al., 2015). In the present study, the specific activities of T-SOD, CuZn-SOD, Mn-SOD and MDA contents were not significantly affected by allicin supplementation, while the specific activity of T-AOC in larvae fed diets with 0.005, 0.01, and 0.02% allicin was significantly higher than the control. Moreover, the activity of CAT in larvae fed the diet with 0.02% allicin was significantly higher than the other three dietary treatments. These results indicated that the suitable supplementation of allicin could increase antioxidant capacity to some extent. However, SOD activity and MDA content were not significantly affected, which was probably due to different species, sizes, experimental conditions. Therefore, the effect of allicin on oxidative stress of larvae needs further study.

High mortality was one of the important limiting factors during fish larvae culture, and more attention has been paid to enhance larval health to improve the survival rate (Rojo-Cebreros et al., 2018). Allicin has been used as immunostimulants to enhance the immune response in mice (Bruck et al., 2005), chicken (Wang et al., 2017), pig (Lan et al., 2017) and many fish species (Nya and Austin, 2011; Abdel-Daim et al., 2015; Xu et al., 2020). NO is a key molecule in the immune-neuroendocrine integration cooperating with other molecules in vertebrates (Pederzoli and Mola, 2016), and showed protective roles of immuno-protection against pathogenic infection via regulating Akt and ERK pathway (Zhou et al., 2014). In the present study, both the content of NO and the activity of NOS increased significantly with dietary allicin increasing from 0 to 0.005%, and then showed a trend to decrease with further increasing allicin dosage. The increased NO content and NOS activity also supported the immuno-stimulant functions of allicin in fish larvae. Besides the NO, the inflammatory response was induced during pathogenic infection and other environmental stress. Allicin has also been found to have anti-inflammatory effects in mammalian studies (El-Sheakh et al., 2016; Panyod et al., 2016; Abdel-Daim et al., 2017; Shi et al., 2017; Li et al., 2020). In the present study, transcriptional levels of *cox-2*, *il-1*β and *il-6* significantly decreased with supplementation of allicin. Similarly, Arellano Buendia et al. (2018) had revealed that the allicin treatment decrease proinflammatory cytokines (*il-1*β and *il-6*) showing the same effect on both systemic and tissue levels of diabetic rats. Moreover, both *in vitro* and *in vivo*, allicin has been reported to modulate the production of *il-1*β, *il-6* and *tnfα* at both the mRNA and protein levels (Alam et al., 2018; Qian et al., 2018). Therefore, these results suggested that allicin supplementation could enhance immunity and alleviate inflammation of large yellow croaker larvae.

Besides fish survival, the sufficient growth with suitable feeding is also important for fish larvae aquaculture. During the fish larval stage, orexigenic stimulation plays a critical role in individual viability (Opazo et al., 2018). Food intake in fish is regulated by both brain factors such as neuropeptide Y (NPY), cocaine and amphetamine-regulated transcript (CART) and anorexigenic proopio melanocortin (POMC), and peripheral factors such as ghrelin and leptin (Ronnestad et al., 2017; Volkoff, 2019). Especially, ghrelin can activate NPY neurons and inhibit POMC/CART neurons in the arcuate nucleus (ARC), resulting in increased appetite (Chen et al., 2004). Conversely, leptin suppresses appetite by inhibiting NPY neurons and activating POMC/CART neurons (Robertson et al., 2008). In the present study, we found that the transcriptional levels of *npy* and *ghrelin* increased with fasting time extending and reached a peak after 24-h, while *leptin* expression firstly increased and then decreased with the prolongation of fasting time. These results indicated that *npy* and *ghrelin* were food intake stimulatory while *leptin* was food intake inhibitory in the larval stage of large yellow croaker. Similar results had also been reported in other fish during fasting, such as *npy* expression in goldfish (Narnaware et al., 2000), coho salmon (Silverstein et al., 1998) and yellowtail (Hosomi et al., 2014), *ghrelin* expression in sea bass (Terova et al., 2008), zebrafish (Amole and Unniappan, 2009) and grass carp (Feng et al., 2013), *leptin* expression in northern snakehead (Wen et al., 2020). More importantly, the transcriptional level of *npy* in larvae fed the diet with 0.01% allicin was investigated and was found to be significantly higher than the control group after fasting for 6-h and 24-h. The result suggested that dietary allicin supplementation significantly increased the appetite of large yellow croaker. Additionally, compared with the control group, the transcriptional level of *leptin* showed no significant difference in fish fed the diet with 0.005 and 0.01% allicin, but significantly increased 0.02% allicin after 6-h and 24-h fasting. The results suggested that supplementation of too high concentrations of allicin leads to the inhibition of fish larvae appetite. Therefore, these results suggested that dietary allicin supplementation might promote the appetite of large yellow croaker larvae at an appropriate dosage but inhibit the appetite of large yellow croaker larvae at too high dosage.

In summary, the present study showed that appropriate supplementation (0.005–0.01%) of allicin could promote the growth and survival of large yellow croaker larvae through promoting intestinal development, alleviating inflammation and enhancing appetite. Further studies are needed to illustrate the inner regulatory mechanisms of fish survival and growth by allicin, involving the cell signaling pathways.

## Data Availability Statement

The raw data supporting the conclusions of this article will be made available by the authors, without undue reservation.

## Ethics Statement

The animal study was reviewed and approved by Chinese Order No. 676 of the State Council.

## Author Contributions

KM, QA, and WH designed the research. WH, YL, and CY conducted the research. WH, NX, and ZY analyzed the data. WH wrote the manuscript. WX and YM provided language help. All authors reviewed and approved the final manuscript.

## Conflict of Interest

The authors declare that the research was conducted in the absence of any commercial or financial relationships that could be construed as a potential conflict of interest.
